# Perfusion gradients promote delayed perihaematomal oedema in intracerebral haemorrhage

**DOI:** 10.1093/braincomms/fcad133

**Published:** 2023-04-24

**Authors:** Enrico Fainardi, Giorgio Busto, Elisa Scola, Ilaria Casetta, Katsuhiro Mizutani, Arturo Consoli, Gregoire Boulouis, Alessandro Padovani, Andrea Morotti

**Affiliations:** Neuroradiology Unit, Department of Experimental and Clinical Biomedical Sciences ‘Mario Serio’, University of Florence, Florence 50134, Italy; Neuroradiology Unit, Department of Radiology, Careggi University Hospital, Florence 50139, Italy; Neuroradiology Unit, Department of Radiology, Careggi University Hospital, Florence 50139, Italy; Section of Neurological, Psychiatric and Psychological Sciences, Department of Biomedical and Specialist Surgical Sciences, University of Ferrara, Ferrara 44121, Italy; Department of Neurosurgery, Keio University School of Medicine, Tokyo 160-8582, Japan; Department of Interventional and Diagnostic Neuroradiology, Hopitâl Foch, Suresnes, le de France, Paris 92150, France; Department of Neuroradiology, University Hospital of Tours, Centre Val de Loire Region, Tours 37020, France; Department of Clinical and Experimental Sciences, Neurology Unit, University of Brescia, Brescia 25121, Italy; Department of Neurological Sciences and Vision, Neurology Unit, ASST Spedali Civili, Brescia 25123, Italy; Department of Neurological Sciences and Vision, Neurology Unit, ASST Spedali Civili, Brescia 25123, Italy

**Keywords:** CT perfusion, intracerebral haemorrhage, perihaematomal oedema, perfusion and hydrostatic pressure gradients

## Abstract

Perihaematomal oedema is a potential therapeutic target to improve outcome of patients with intracerebral haemorrhage, but its pathophysiology remains poorly elucidated. We investigated the longitudinal changes of cerebral perfusion and their influence on perihaematomal oedema development in 150 patients with intracerebral haemorrhage who underwent computed tomography perfusion within 6 h from onset, at 24 h and at 7 days. Perfusion parameters were measured in haemorrhagic core, perihaematomal rim, surrounding normal appearing and contralateral brain tissue. Computed tomography perfusion parameters gradually improved from the core to the periphery in each time interval with an early increase at 24 h followed by a delayed decline at 7 days compared with admission values (*P* < 0.001). Multivariable linear regression analysis showed that haematoma volume and cerebral blood flow gradient between normal appearing and perihaematomal rim were independently associated with absolute perihaematomal oedema volume in the different time points (within 6 h, *B* = 0.128, *P* = 0.032; at 24 h, *B* = 0.133, *P* = 0.016; at 7 days, *B* = 0.218, *P* < 0.001). In a secondary analysis with relative perihaematomal oedema as the outcome of interest, cerebral blood flow gradient between normal appearing and perihaematomal rim was an independent predictor of perihaematomal oedema only at 7 days (*B* = 0.239, *P* = 0.002). Our findings raise the intriguing hypothesis that perfusion gradients promote perihaematomal oedema development in the subacute phase after intracerebral haemorrhage.

## Teaser

Fainardi *et al.* investigated the longitudinal change of perfusion parameters and their association with oedema in patients with acute intracerebral haemorrhage. Perihaemorrhagic perfusion gradients promote oedema development and might represent a novel therapeutic target in the subacute phase following intracerebral haemorrhage.

## Graphical Abstract

**Graphical Abstract:**
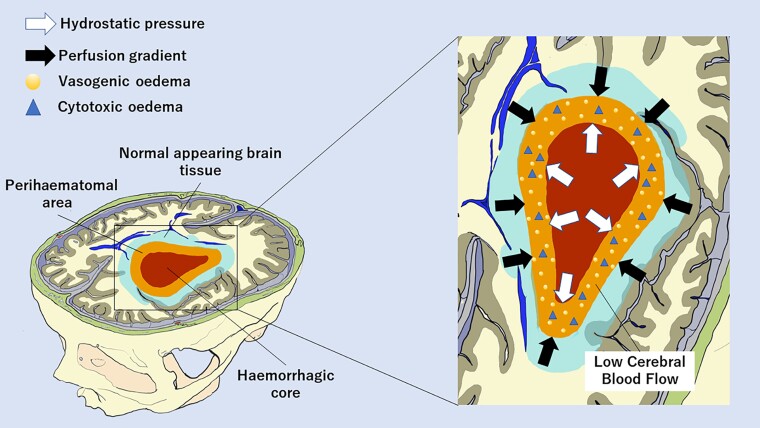

## Introduction

Spontaneous intracerebral haemorrhage (ICH) is a devastating cerebrovascular disease that accounts for up to 20% of all strokes and is characterized by high disability and mortality rates.^1^ Primary brain damage occurs because of the mass effect of the haemorrhage, and ICH volume is the strongest prognostic determinant. However, other biological mechanisms contribute to secondary brain injury such as perihaematomal oedema (PHO) arising from thrombin release, inflammatory response, blood–brain barrier (BBB) breakdown and toxic effects of haemoglobin degradation products.^2^ Therefore, PHO might represent an appealing treatment target. It is well known that PHO develops rapidly after bleeding and peaks at 24 h and at 14–21 days from onset, but the pathophysiology and determinants of PHO remain complex and controversial.^3^ In particular, the relationship between brain perfusion and PHO development is still poorly characterized.^3^ Inconclusive results were obtained investigating the association between PHO volume, ICH enlargement and prognosis due to the close relationship between the haematoma and PHO volumes.^3^ On the other hand, advanced neuroimaging techniques, such as positron emission tomography, magnetic resonance diffusion-weighted and perfusion-weighted imaging and computed tomography perfusion (CTP), were not able to fully elucidate the natural history of perihaematomal tissue. Previous studies observed a moderate hypoperfusion suggesting vasogenic oedema and hypometabolism,^4–8^ in the context of a concentric distribution of perfusion parameters gradually improving from the core to the periphery.^8^ Conversely, a subsequent DWI study demonstrated the presence of perihaematomal cytotoxic oedema at 7 days after bleeding in a subgroup of patients.^9^ In addition, recent publications based on CTP revealed that the risk of haematoma expansion was higher in ICH patients with perihaematomal low cerebral blood volume (CBV) values at 24 h after onset,^10^ whereas a poor outcome was more likely in subjects with reduced perihaematomal cerebral blood flow (CBF) levels at 24 h^11^ and even more strongly at 7 days after ICH.^12^ As these findings indicate a potential role of hypoperfusion in PHO development and longitudinal evolution, we explored the temporal changes in CTP parameters within and around the haematoma and their association with the extent of PHO after ICH at different time points.

## Patients and methods

All the study procedures were approved by the Institutional Review Board of the Azienda Ospedaliera Universitaria, Arcispedale S. Anna, Ferrara, Italy, and clinical information was recorded during routine clinical activity. Written informed consent was obtained from each patient or from his/her legal representatives in the setting of a prospective study investigating longitudinal CTP parameters in patients with acute ICH.^8^

### Patient characteristics and assessment

This was a retrospective analysis conducted on a prospectively collected cohort of spontaneous ICH patients admitted at a single academic hospital from January 2010 to November 2015 meeting the following inclusion criteria: (i) supratentorial ICH on admission non-contrast CT (NCCT) scans performed within 6 h from symptom onset or time last seen well; (ii) availability of baseline and follow-up NCCT and CTP studies completed at the three established time points (admission, 24 h and 7 days); and (iii) age > 18 years. Exclusion criteria were as follows: (i) infratentorial ICH; (ii) ICH secondary to tumour, trauma, coagulopathy, aneurysms, vascular malformations or haemorrhagic transformation of brain infarction; (iii) ICH surgical evacuation performed before follow-up NCCT; (iv) anticoagulant treatment with vitamin K antagonists and international normalized ratio > 1.5 or treatment with direct oral anticoagulants or other known coagulopathy; (v) pregnancy; (vi) absolute contraindication to the administration of iodinated contrast material; (vii) poor quality of CT acquisition due to motion artefacts; and (viii) inability to complete baseline and follow-up NCCT and CTP protocol. None of the included patients was mechanically ventilated or sedated, and none received an intracranial pressure monitoring. Age, sex, hypertension, antiplatelet treatment, admission blood pressure (BP) and time from onset to baseline NCCT were collected. Disease severity on admission was measured with the National Institutes of Health Stroke Scale (NIHSS). Functional outcome was assessed using the modified Rankin Scale (mRS) at 3 months and mRS ≤ 2 was considered good functional outcome. All patients received BP treatment in the acute phase according to the American Heart Association/American Stroke Association guidelines.^13^

### Imaging acquisition, processing and analysis

**Figure 1:**
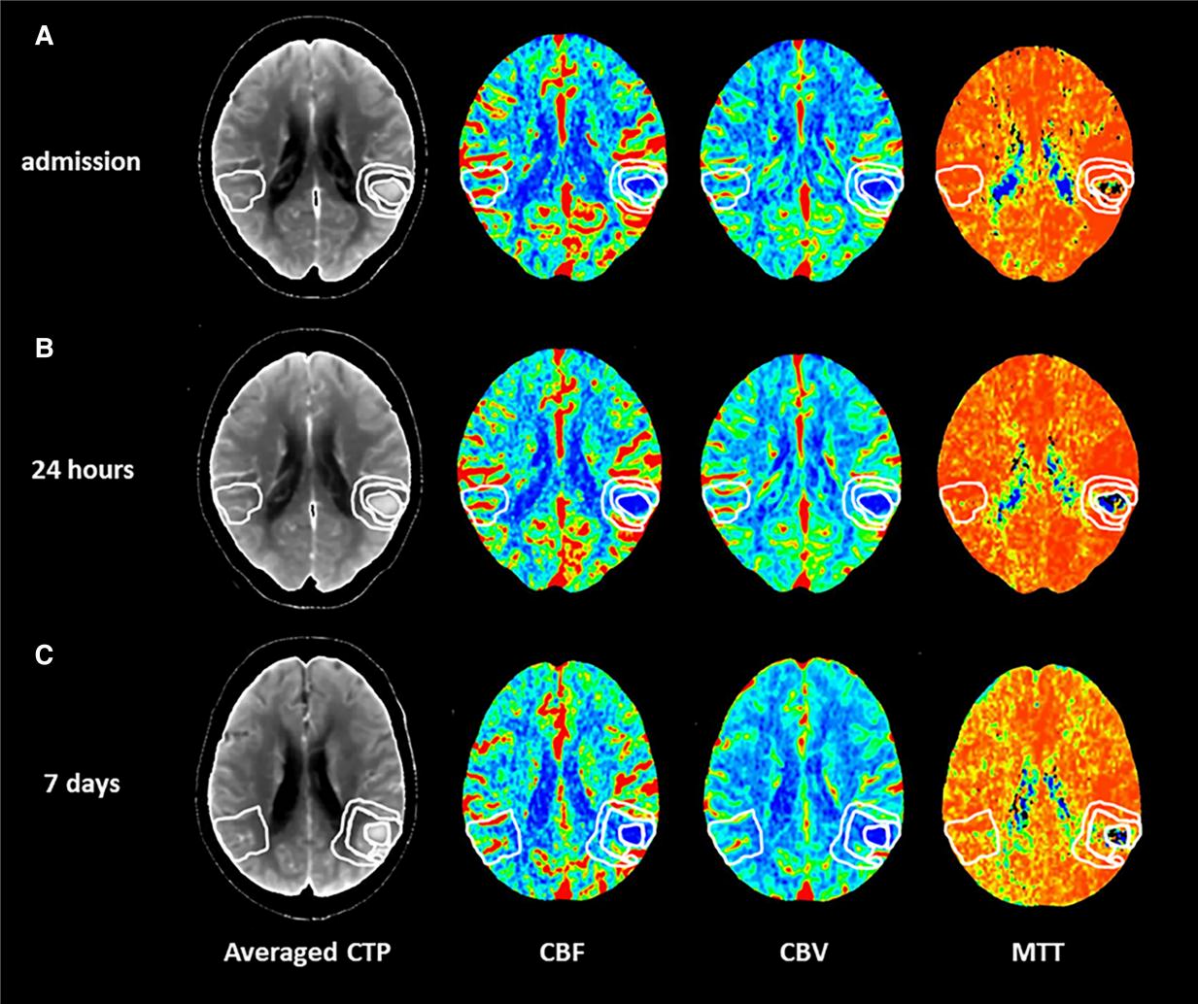
**Perfusion mapping of acute spontaneous intracerebral haemorrhage.** Computed tomography perfusion in a patient with acute spontaneous intracerebral haemorrhage located in the left temporal and parietal lobes performed at admission (**A**), at 24 h (**B**) and at 7 days (**C**) on averaged computed tomography perfusion images (averaged CTP). CBF, cerebral blood flow; CBV, cerebral blood volume; MTT, mean transit time.

All imaging was conducted on 64-slice scanners (GE Healthcare, Waukesha, WI, USA). NCCT and CTP studies was performed at admission, (*T*_0_), within 24 h (*T*_1_) and at 7 days (*T*_7_) and co-registered. In all patients, NCCT scan was immediately followed by CTP examination. Haematoma location was evaluated on baseline NCCT and classified as deep (bleeding involving the thalamus, basal ganglia, internal capsule or deep periventricular white matter) and lobar (bleeding affecting the cortex and the cortical–subcortical junction of one or more lobes). Presence of intraventricular haemorrhage (IVH) on baseline NCCT was also recorded. Haematoma and absolute perihaematomal oedema (aPHO) volumes were calculated on NCCT with a semi-automated computer-assisted planimetric measurement with ITK-SNAP 3.8.0 software. Relative perihaematomal oedema (rPHO) volume was defined as aPHO volume divided by haematoma volume as previously described.^14^ CTP studies were performed with a dynamic first-pass bolus-tracking methodology according to a one-phase imaging protocol. CBF, CBV and mean transit time (MTT) maps were generated using a commercially available delay-sensitive deconvolution software (CT Perfusion 3, GE Healthcare, Waukesha, WI, USA). CBF, CBV and MTT values were expressed in mL/100 g/min, mL/100 g and seconds, respectively. Average CTP maps were created by averaging the cine (dynamic) CTP source images over the duration of the first pass of contrast. These average CTP images were used to exclude cerebrospinal fluid and skull from analysis using Hounsfield unit thresholds. Large blood vessels were automatically excluded from calculation by the software. As shown in Fig. 1, CBF, CBV and MTT levels were measured in four different regions of interest (ROIs) >1 cm^2^ and drawn freehand by two neuroradiologists with >10-year experience (ES and EF) on averaged CTP images in every section with evidence of bleeding: (i) haemorrhagic core (HC); (ii) perihaematomal rim (PR): the low density area located around the clot reflecting oedema development; (iii) normal appearing (NA): 1-cm rim of NA brain tissue on NCCT, surrounding the perilesional rim; (iv) contralateral (CO): an area mirroring the region including the clot and the perihaematomal rim located in the CO hemisphere. A good inter-rater reliability interclass correlation (ICC) > 0.80 for ROI determination was observed in a subset of 15 patients. In the same subset, intra and inter-rater reliability was tested between two imaging raters (G.B., neuroradiologist with <10-year experience and EF, neuroradiologist with >10-year experience in ICH imaging) for determination of ICH volume, PHO volume and CTP parameters, with evidence of good agreement (ICC > 0.80 for all imaging parameters). Absolute differences in perfusion parameters (ΔCBF, ΔCBV and ΔMTT) from NA to PR (NA-PR) and from PR to HC (PR-HC), representing the perfusion gradients between NA and PR and between PR and HC, were also calculated in all the three time points of the study. All the images were analysed by a neuroradiologist with >10-year experience in CTP acquisition and interpretation. The imaging raters were blinded and in particular to CTP images timing.

### Statistical analysis

Data distribution was checked with Kolmogorov–Smirnov test for normality. Continuous variables were expressed as median [interquartile range (IQR)] and compared with Friedman test. Categorical variables were expressed as count (percentage). Spearman test was used to explore correlations between continuous variables. aPHO was the main outcome of interest of the analysis. Independent predictors of aPHO were explored through linear regression with backward elimination at *P* < 0.1. aPHO was log-transformed in linear regression models, including the following variables: age, sex, SBP, ICH volume and location, presence of IVH and CTP parameters (absolute values and absolute changes). To avoid multicollinearity, different CTP parameters were not included together in the same linear regression models, and the main analyses were focused on CBF. In a secondary analysis, linear regression models were performed with rPHO as the outcome of interest. Finally, all regression models were repeated adjusting for multiple testing with the false discovery rate method, as previously described by Benjamini and Hochberg.^15^ All the analyses were performed with the statistical software SPSS 21.0 (www.spss.com), and statistical significance was set at *P* values < 0.05.

## Results

**Figure 2:**
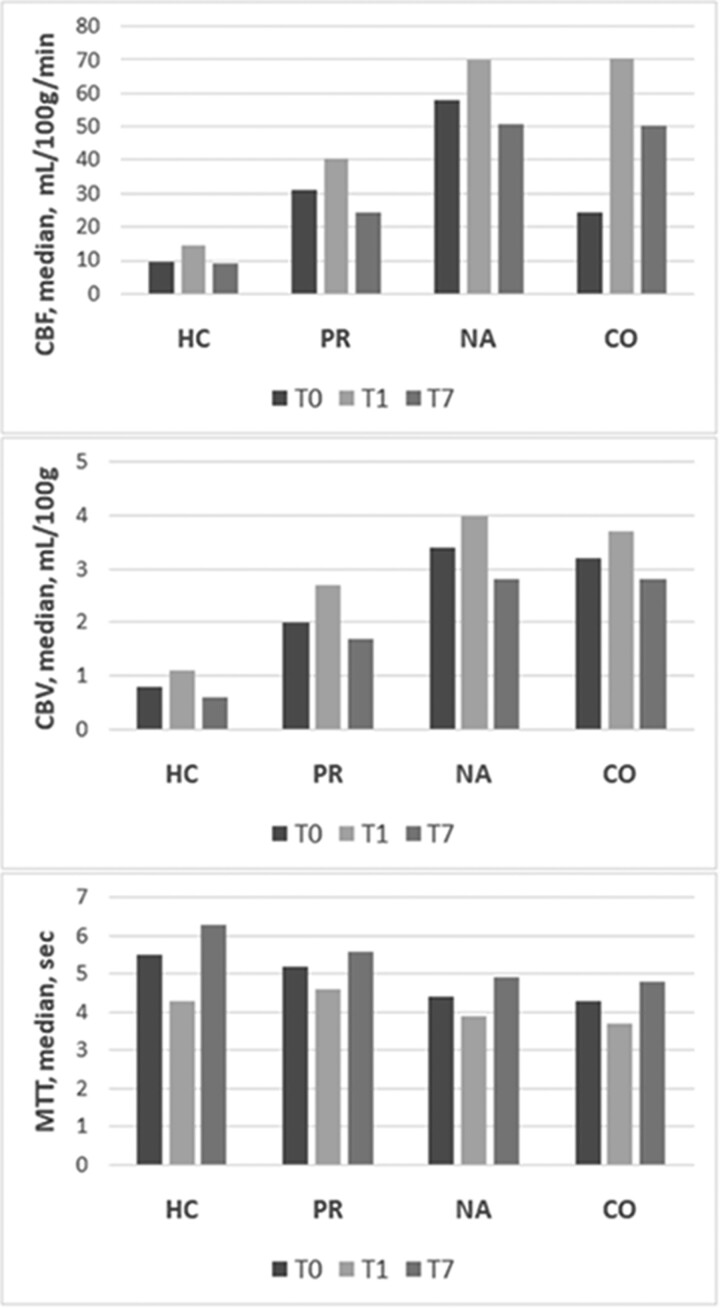
**Longitudinal evolution of CTP parameters.** CBF, CBV and MTT in HC, PR, NA tissue and CO area measured at admission, (*T*_0_), within 24 h (*T*_1_) and at 7 days (*T*_7_). CBF, cerebral blood flow; CBV, cerebral blood volume; CO, contralateral; HC, haemorrhagic core; MTT, mean transit time; NA, normal appearing; PR, perihaemorrhagic rim.

A total of 223 patients with primary spontaneous ICH were identified, and 73 subjects were excluded based on predefined exclusion criteria (41 had secondary ICH or coagulopathy, 25 had contraindications or inability to complete the entire imaging protocol, and 7 underwent surgical haematoma evacuation). Excluded patients had larger ICH volume, lower GCS and higher mortality at 90 days. Overall, 150 patients met study eligibility criteria. Table 1 summarizes the characteristics of the study population. Table 2 shows the longitudinal evolution of ICH volume, PHO and perfusion parameters over time in the first week after ICH onset. Compared with baseline values, ICH volume increased at 24 h and then decreased, but with size greater than admission, at 7 days post-bleeding (*P* < 0.001), aPHO and rPHO volumes gradually increased at 24 h and 7 days after ICH (*P* < 0.001), whereas CBF and CBV increased at 24 h and declined at 7 days and MTT decreased at 24 h and was more prolonged at 7 days after bleeding in HC, PR, NA and CO (*P* < 0.001). Table 3 reports the comparison between perfusion values across different ROIs. CBF, CBV and MTT were concentrically distributed and gradually improved from the core to the periphery in each time interval examined in which NA and CO had similar values. The longitudinal changes in CTP parameters across the four brain ROIs are represented in Fig. 2. Correlations of aPHO volume with ICH volume and CBF within and around the haematoma over time are illustrated in Table 4. ICH volume had the strongest positive correlation with aPHO volume in all the three time points evaluated (*P* < 0.001). A significant inverse correlation with aPHO volume was also found for CBF in PR (*P* < 0.001) and, to a lesser extent, in HC (*T*_0_, *P* = 0.004; *T*_1_, *P* = 0.002; and *T*_7_, *P* = 0.010), whereas this association was not statistically significant for CBF in NA. A significant inverse association with aPHO volume was observed for ΔCBF PR-HC in *T*_0_ (*P* < 0.002), *T*_1_ (*P* < 0.001) and *T*_7_ (*P* < 0.003), while ΔCBF NA-PR was positively correlated with aPHO in *T*_0_ (*P* < 0.003), in *T*_7_ (*P* < 0.002), but not in *T*_1_. Weaker and similar relationships with aPHO volume were obtained after the analysis of CBV and MTT timing in HC, PR and NA (Supplementary Tables 1 and 2). As summarized in Table 5, multivariable linear regression analysis showed that only ICH volume and CBF gradients between NA and PR were independently associated with aPHO volume in all the three time points assessed. In particular, the association between ΔCBF NA-PR and aPHO volume was stronger at 7 days (*T*_0_, *B* = 0.128, *P* = 0.032; *T*_1_, *B* = 0.133, *P* = 0.016; and *T*_7_, *B* = 0.218, *P* < 0.001). In a secondary analysis with rPHO volume as the main outcome of interest, ΔCBF NA-PR was an independent predictor of rPHO volume only at 7 days (*T*_0_, *B* = 0.099, *P* = 0.221; *T*_1_, *B* = 0.105, *P* = 0.181; and *T*_7_, *B* = 0.239, *P* = 0.002). All our findings remained statistically significant after adjusting for multiple testing.

**Table 1:** Cohort characteristics

	All
	*n* = 150
Age, median (IQR), y	68 (61–74)
Sex, male, *n* (%)	71 (47.3)
History of hypertension, *n* (%)	92 (61.3)
Antiplatelet treatment, *n* (%)	42 (28.0)
SBP, median (IQR), mmHg	150 (130–170)
DBP, median (IQR), mmHg	80 (80–91)
NIHSS, median (IQR)	14 (10–19)
Time from onset to NCCT, median (IQR)h	3.1 (2.4–3.8)
Baseline ICH volume, mL	12 (6–19)
ICH location, deep, *n* (%)	91 (60.7)
Presence of IVH, *n* (%)	37 (24.7)
mRS 3–6 at 3 months, *n* (%)	52 (34.7)

DBP, diastolic blood pressure; ICH, intracerebral haemorrhage; IQR, interquartile range; IVH, intraventricular haemorrhage; mRS, modified Rankin Scale; NCCT, non-contrast computed tomography; NIHSS, National Institute of Health Stroke Scale; SBP, systolic blood pressure.

**Table 2:** Temporal evolution of ICH, PHO volumes and perfusion parameters

	*T*_0_	*T*_1_	*T*_7_	*P* (Friedman)
ICH volume, median (IQR), mL	12.4 (5.6–18.5)	15.6 (6.8–25.3)	13.6 (5.6–22.6)	<0.001
aPHO volume, median (IQR), mL	20.5 (10.9–32.4)	23.3 (15.7–46.0)	28.1 (16.2–52.5)	<0.001
rPHO volume, median (IQR), mL	1.8 (1.2–2.7)	1.7 (1.2–2.7)	2.1 (1.5–3.1)	<0.001
HC CBF, median (IQR), mL/100 g/min	9.3 (6.0–13.5)	14.3 (9.4–20.5)	9.1 (5.3–11.5)	<0.001
PR CBF, median (IQR), mL/100 g/min	30.9 (21–47.6)	40.4 (30.1–58–2)	24.2 (17.3–40.3)	<0.001
NA CBF, median (IQR), mL/100 g/min	58.1 (43.4–77.3)	70.0 (47.2–94.4)	50.6 (37.3–69.3)	<0.001
CO CBF, median (IQR), mL/100 g/min	59.4 (41.9–76.8)	70.6 (46.8–95.0)	50.4 (36.2–68.1)	<0.001
HC CBV, median (IQR), mL/100g	0.8 (0.5–1–1)	1.1 (0.7–1.6)	0.6 (0.4–0.8)	<0.001
PR CBV, median (IQR), mL/100g	2.0 (1.4–3.0)	2.7 (2.0–3.4)	1.7 (1.0–2.6)	<0.001
NA CBV, median (IQR), mL/100g	3.4 (2.6–4.2)	4.0 (3.0–5.6)	2.8 (2.1–4.3)	<0.001
CO CBV, median (IQR), mL/100g	3.2 (2.6–4.4)	3.7 (3.2–5.2)	2.8 (2.1–4.3)	<0.001
HC MTT, median (IQR), seconds	5.5 (4.1–6.8)	4.3 (3.3–5.6)	6.3 (4.5–7.5)	<0.001
PR MTT, median (IQR), seconds	5.2 (4.5–6–6)	4.6 (3.7–5.7)	5.6 (4.9–7.0)	<0.001
NA MTT, median (IQR), seconds	4.4 (3.6–5.2)	3.9 (3.0–4.6)	4.9 (4.1–5.7)	<0.001
CO MTT, median (IQR), seconds	4.3 (3.4–4.9)	3.7 (2.8–4.4)	4.8 (3.8–5.4)	<0.001

aPHO, absolute PHO; CBF, cerebral blood flow; CBV, cerebral blood volume; CO, contralateral hemisphere; HC, haemorrhagic core; MTT, mean transit time; NA, normal appearing brain tissue; PR, perihaematomal rim; rPHO, relative PHO; *T*_0_, admission; *T*_1_, 24 h after bleeding; *T*_7_, 7 days after bleeding.

**Table 3:** Topographic distribution of perfusion parameters in the different time points

	HC	PR	NA	CO	*P* (Friedman)
CBF *T*_0_, median (IQR), mL/100 g/min	9.3 (6.0–13.5)	30.9 (21–47.6)	58.1 (43.5–76.8)	59.4 (41.9–76.8)	<0.001
CBF *T*_1_, median (IQR), mL/100 g/min	14.3 (9.4–20.5)	40.4 (30.1–58–2)	70.0 (47.2–94.4)	70.6 (46.8–95.0)	<0.001
CBF *T*_7_, median (IQR), mL/100 g/min	9.1 (5.3–11.5)	24.2 (17.3–40.3)	50.6 (37.3–69.3)	50.4 (36.2–68.1)	<0.001
CBV *T*_0_, median (IQR), mL/100g	0.8 (0.5–1–1)	2.0 (1.4–3.0)	3.4 (2.6–4.2)	3.2 (2.6–4.4)	<0.001
CBV *T*_1_, median (IQR), mL/100g	1.1 (0.7–1.6)	2.7 (2.0–3.4)	4.0 (3.0–5.6)	3.7 (3.2–5.2)	<0.001
CBV *T*_7_, median (IQR), mL/100g	0.6 (0.4–0.8)	1.7 (1.0–2.6)	2.8 (2.1–4.3)	2.8 (2.1–4.3)	<0.001
MTT *T*_0_, median (IQR), seconds	5.5 (4.1–6.8)	5.2 (4.5–6–6)	4.4 (3.6–5.2)	4.3 (3.4–4.9)	<0.001
MTT *T*_1_, median (IQR), seconds	4.3 (3.3–5.6)	4.6 (3.7–5.7)	3.9 (3.0–4.6)	3.7 (2.8–4.4)	<0.001
MTT *T*_7_, median (IQR), seconds	6.3 (4.5–7.5)	5.6 (4.9–7.0)	4.9 (4.1–5.7)	4.8 (3.8–5.4)	<0.001

CBF, cerebral blood flow; CBV, cerebral blood volume; CO, contralateral hemisphere; HC, haemorrhagic core; MTT, mean transit time; NA, normal appearing brain tissue; PR, perihaematomal rim; *T*_0_, admission; *T*_1_, 24 h after bleeding; *T*_7_, 7 days after bleeding.

**Table 4:** Correlations between aPHO volume, ICH volume and CBF within and around the haematoma at different time points

	aPHO *T*_0_		aPHO *T*_1_		aPHO *T*_7_	
	Rho	*P*	Rho	*P*	Rho	*P*
ICH volume	0.758	<0.001	0.866	<0.001	0.862	<0.001
HC CBF	−0.233	0.004	−0.254	0.002	−0.209	0.010
PR CBF	−0.294	<0.001	−0.319	<0.001	−0.343	<0.001
NA CBF	0.020	0.809	−0.051	0.536	0.040	0.629
ΔCBF NA-PR	0.244	0.003	0.008	0.217	0.247	0.002
ΔCBF PR-HC	−0.256	0.002	−0.273	0.001	−0.245	0.003

aPHO, absolute perihaematomal oedema; CBF, cerebral blood flow; CO, contralateral hemisphere;

HC, haemorrhagic core; ICH, intracerebral haemorrhage; NA, normal appearing brain tissue; PR, perihaematomal rim; Rho, Spearman Rank Correlation; *T*_0_, admission; *T*_1_, 24 h after bleeding; *T*_7_, 7 days after bleeding; ΔCBF NA-PR, absolute changes in CBF from NA to PR; ΔCBF PR-HC, absolute changes in CBF from PR to HC.

**Table 5:** Multivariable predictors of aPHO volume at different time points

	aPHO T_0_	
	B	*P*
ICH volume	0.621	<0.001
Presence of IVH	0.158	0.017
ΔCBF NA-PR	0.128	0.032
	**aPHO *T*_1_**	
	**B**	***P***
ICH volume	0.762	<0.001
ΔCBF NA-PR	0.133	0.016
	**aPHO *T*_7_**	
	**B**	***P***
ICH volume	0.687	<0.001
IVH presence	0.112	0.068
ΔCBF NA-PR	0.218	<0.001

Linear regression with backward elimination at *P* < 0.1. Variables entered into the model: age, sex, systolic blood pressure, intracerebral haemorrhage (ICH) location, ICH volume, IVH presence, haemorrhagic core cerebral blood flow (HC CBF), perihaematomal rim CBF (PR CBF) normal appearing CBF (NA CBF), absolute difference in CBF from NA to PR (ΔCBF NA-PR) and absolute difference in CBF from PR to HC (ΔCBF PR-HC); B, beta coefficient.

## Discussion

**Figure 3:**
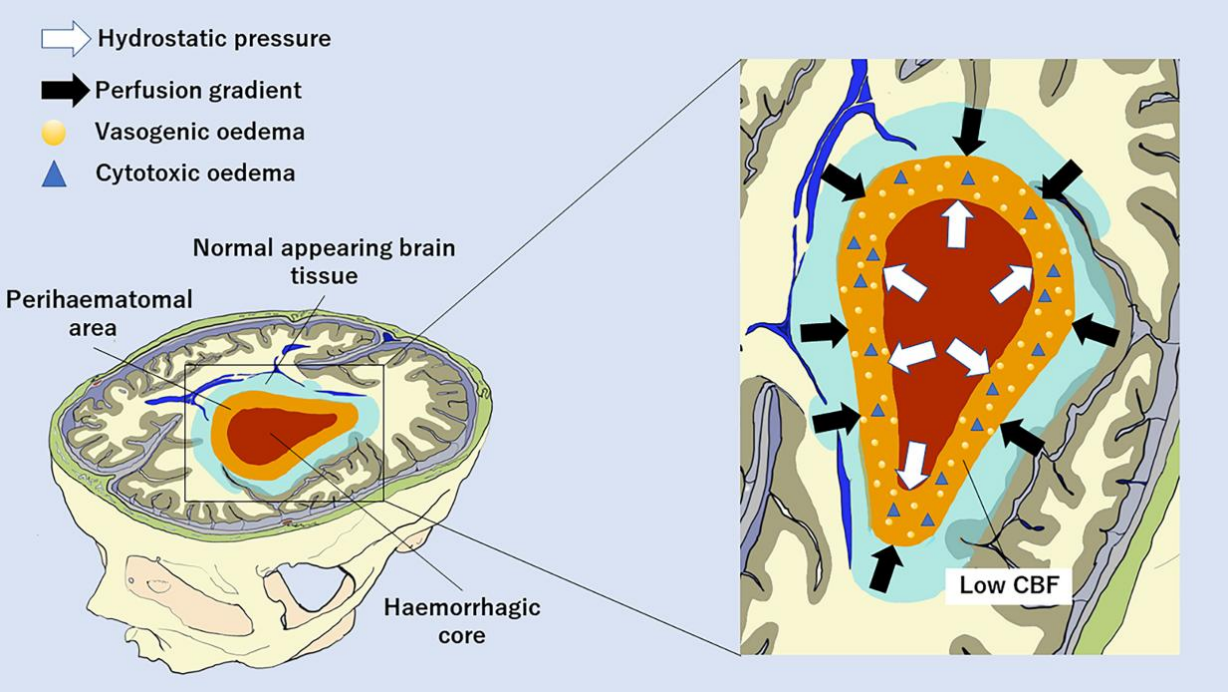
**Hypothetical hydrostatic and perfusion factors promoting delayed PHO at 7 days after intracerebral haemorrhage.** The hydrostatic pressure gradient between the HC and perihaematomal area is due to the high pressure of the clot that is not counteracted by the low pressure of the perihaematomal area. The perfusion gradient between NA brain tissue and perihaematomal area is created by normal CBF levels of non-injured tissue opposed to low perihaemorragic CBF values. Both mechanisms contribute to water migration from intravascular to extracellular spaces. This is facilitated by also the BBB breakdown caused by the extension of perihaematomal cytotoxic oedema to vessel wall endothelial cells. CBF, cerebral blood flow.

We have described the longitudinal evolution of brain perfusion after acute ICH and the relationship between perfusion gradients and PHO. All perfusion parameters showed a biphasic profile with an early perfusion improvement at 24 h followed by a delayed decline at 7 days compared with admission values measured within 6 h from onset. This pattern was observed not only in the HC and perihaematomal area but also in NA brain tissue and in CO hemisphere, suggesting that the haemodynamic response occurring after an acute ICH is generalized to the whole brain. We also observed that the centrifugal distribution of CTP parameters with a gradual increase from the core to the periphery persisted during the transition from acute to subacute ICH phase, in line with a previous study.^8^ Of note, while in perihaematomal oedematous region the initial hypoperfusion in part recovered at 24 h and then became more pronounced at 7 days, as recently suggested,^11,12^ in the unaffected ipsilateral and CO brain tissue, we observed perfusion values consistent with hyperaemia/hyperperfusion at 24 h and a perfusion normalization at 7 days.^8^ Therefore, our data partially resembled the acute hibernation and the subacute reperfusion phases previously identified at the level of perihaematomal rim.^16^ In this setting, we documented that the centrifugal distribution of CTP parameters with a gradual increase from the core to the periphery, already reported in the acute stage of ICH,^8^ persisted during the transition from acute to subacute ICH phases. However, the main finding of this study was the demonstration that ΔCBF NA-PR was independently associated with aPHO volume on admission, at 24 h and 7 days after bleeding, along with ICH volume. This relationship was stronger at 7 days and ΔCBF NA-PR was the only independent predictor of rPHO volume. Our results suggest that the perfusion gradient between NA brain tissue located around the injured region and perihaematomal oedematous area contributes to PHO development in the acute and even more in the subacute stages of ICH. This observation might be an epiphenomenon of ICH size, with larger haemorrhages having a higher extent of PHO. Nevertheless, our findings do not support this hypothesis, as ICH volume and perfusion gradients were both associated with PHO, independently from each other. The combination of these two factors seems to promote principally delayed PHO formation at 7 days when ICH size is still large and perihaematomal hypoperfusion increase,^12^ favouring the creation of a more prominent ΔCBF NA-PR. The precise mechanisms underlying this process remain speculative, and our findings are best interpreted as hypothesis generating. The haematoma mass effect could induce PHO formation by generating a hydrostatic pressure gradient between HC and PR due to the high pressure of the clot that is not counteracted by the low pressure of PHO.^3,17,18^ This gradient might represent the main driving force leading to water migration from the intravascular compartment to the extracellular space, in presence of a breakdown of the BBB.^2,17^ Perfusion gradient between NA and PR may trigger PHO development acting as a hydrostatic force as well, with the concurrence of an increase in the amount cytotoxic oedema.^9^ In fact, as also documented in ischaemic oedema,^19^ it is possible that cytotoxic oedema extends to vessel wall endothelial cells leading to a further opening of BBB that results in water and protein extravasation and formation of an additional osmotic gradient.^17,18^ However, reverse causality might also explain our findings, with PHO being a determinant and not a consequence of perfusion changes after ICH. A hypothetical interplay between perfusion and hydrostatic pressure gradients is depicted in Fig. 3. Our findings might have implications for future studies. While most of the potential therapeutic targets in acute ICH are strongly time dependent with a narrow treatment window, such as haematoma expansion, PHO might be a potentially modifiable prognostic determinant, with a longer time window for treatment. In fact, we showed that the relationship between perihaematomal perfusion and PHO was stronger outside the hyperacute phase, in particular at 7 days after bleeding. Furthermore, haematoma clearance after ICH appears a plausible biologic target to improve functional outcome and further research focused on the interaction between haematoma clearance and perihaemorrhagic perfusion appears warranted.^20^ Some limitations of our findings should be acknowledged. First, our findings were obtained from a relatively small sample size collected at a single institution. Second, as this study was based on a retrospective analysis, our findings require prospective validation. Third, temporal variations in BP and cerebral perfusion pressure were not systematically collected and were not available for adjustment in multivariable analyses. Furthermore, reduced cardiac ejection fraction might have influenced our analysis, and we were unable to account for this potential confounder.^21^ However, none of the included subjects had a medical history or clinical symptoms suggestive of heart failure. Fourth, a significant proportion of patients had good functional outcome. This observation and discrepancy with previous literature might be explained by selection bias.^22,23^ Patients from intensive care units were not enrolled and patients who died in the hyperacute phase were excluded because unable to complete the imaging protocol with CTP at 7 days. The characteristics and clinical severity of excluded patients support the possibility of selection bias. Finally, CTP maps were obtained by using a delay-sensitive software, and therefore, we were not able to exclude delay and dispersion of the contrast bolus that could result in underestimation of CBF values.^24^

## Conclusion

In conclusion, we have described the longitudinal evolution of cerebral perfusion in the entire brain of patients with acute ICH and raised the hypothesis that perihaemorrhagic perfusion gradients might play a role in PHO development. Further research efforts appear warranted to confirm our findings and to characterize the underlying biological mechanisms.

## Supplementary Material

fcad133_Supplementary_Data

## Data Availability

The data set may be accessed upon reasonable request to the corresponding author and Institutional Review board approval.

## Abbreviations

aPHO =: absolute perihaematomal oedema
BBB =: blood–brain barrier
BP =: blood pressure
CBF =: cerebral blood flow
CBV =: cerebral blood volume
CO =: contralateral
CTP =: computed tomography perfusion
HC =: haemorrhagic core
ICC =: interclass correlation
ICH =: intracerebral haemorrhage
IQR =: interquartile range
IVH =: intraventricular haemorrhage
MTT =: mean transit time
NA =: normal appearing
NCCT =: non-contrast CT
NIHSS =: National Institutes of Health Stroke Scale
PHO =: perihaematomal oedema
PR =: perihaematomal rim
ROIs =: regions of interest
rPHO =: relative perihaematomal oedema
