# Supplementary material for: Perfusion gradients promote delayed perihaematomal oedema in intracerebral haemorrhage
Source: Brain Commun. 2023 Apr 24;5(3):fcad133. doi: 10.1093/braincomms/fcad133 (PMC10162681; doi:10.1093/braincomms/fcad133)
Supplement: fcad133_Supplementary_Data [file fcad133_supplementary_data.pdf]

Supplementary Table 1. Correlations between absolute perihaematomal oedema (aPHO) volume and cerebral blood volume (CBV) within and around the haematoma at different time-points.

	**aPHO T0**		**aPHO T1**		**aPHO T7**	
	**Rho**	***P***	**Rho**	***P***	**Rho**	***P***
HC CBV	-0.180	0.027	-0.078	0.343	-0.275	0.001
PR CBV	-0.254	0.002	-0.301	<0.001	-0.279	0.001
NA CBV	0.057	0.489	0.012	0.886	0.046	0.575
ΔCBV NA-PR	0.094	0.252	0.039	0.632	0.247	0.002
ΔCBV PR-HC	-0.162	0.047	-0.225	0.006	-0.245	0.003

Rho, Spearman Rank Correlation; T0, admission; T1, 24 hours after bleeding; T7, 7 days after bleeding; HC, haemorrhagic core; PR, perihematomal rim; NA, normal appearing brain tissue; CO, contralateral hemisphere; ΔCBV NA-PR, absolute changes in CBV from NA to PR; ΔCBV PR-HC, absolute changes in CBV from PR to HC.

Supplementary Table 2. Correlations between absolute perihaematomal oedema (aPHO) volume and mean transit time (MTT) within and around the haematoma at different time-points.

	**aPHO T0**		**aPHO T1**		**aPHO T7**	
	**Rho**	***P***	**Rho**	***P***	**Rho**	***P***
HC MTT	MTT HC	0.135	0.100	0.091	0.269	0.038
PR MTT	MTT PR	0.247	0.002	0.307	<0.001	0.314
NA MTT	MTT NP	0.105	0.202	0.137	0.094	0.234
ΔMTT NA-PR	Δ MTT NP - PR	-0.180	0.027	-0.181	0.027	-0.103
ΔCMTT PR-HC	Δ MTT PR - HC	0.080	0.328	0.153	0.061	0.124

Rho, Spearman Rank Correlation; T0, admission; T1, 24 hours after bleeding; T7, 7 days after bleeding; HC, haemorrhagic core; PR, perihematomal rim; NA, normal appearing brain tissue; CO, contralateral hemisphere; ΔCBV NA-PR, absolute changes in CBV from NA to PR; ΔCBV PR-HC, absolute changes in CBV from PR to HC.
